# Severe Neonatal Epileptic Encephalopathy and *KCNQ2* Mutation: Neuropathological Substrate?

**DOI:** 10.3389/fped.2014.00136

**Published:** 2014-12-19

**Authors:** Charlotte Dalen Meurs-van der Schoor, Mirjam van Weissenbruch, Marjan van Kempen, Marianna Bugiani, Eleonora Aronica, Hanneke Ronner, R. Jeroen Vermeulen

**Affiliations:** ^1^Department of Neonatology, VU University Medical Center, Amsterdam, Netherlands; ^2^Department of Medical Genetics, University Medical Center, Utrecht, Netherlands; ^3^Department of Pathology, Neuroscience Campus Amsterdam, VU University Medical Center, Amsterdam, Netherlands; ^4^Department of Child Neurology, Neuroscience Campus Amsterdam, VU University Medical Center, Amsterdam, Netherlands; ^5^Department of Pathology, Academic Medical Center, Amsterdam, Netherlands; ^6^Department of Clinical Neurophysiology, VU University Medical Center, Amsterdam, Netherlands

**Keywords:** neonatal, neonatal seizures, *KCNQ2* mutation, ion channel gene defect, cortical dysplasia, MRI

## Abstract

**Background:** Neonatal convulsions are clinical manifestations in a heterogeneous group of disorders with different etiology and outcome. They are attributed to several genetic causes.

**Methods:** We describe a patient with intractable neonatal seizures who died from respiratory compromise during a status epilepticus.

**Results:** This case report provides electroencephalogram (EEG), MRI, genetic analysis, and neuropathological data. Genetic analysis revealed a *de novo* heterozygous missense mutation in the *KCNQ2* gene, which encodes a subunit of a voltage-gated potassium channel*. KCNQ2* gene mutation is associated with intractable neonatal seizures. EEG, MRI, data as well as mutation analysis have been described in other *KCNQ2* cases. Post-mortem neuropathological investigation revealed mild malformation of cortical development with increased heterotopic neurons in the deep white matter compared to an age-matched control subject. The new finding of this study is the combination of a *KCNQ2* mutation and the cortical abnormalities.

**Conclusion:** *KCNQ2* mutations should be considered in neonates with refractory epilepsy of unknown cause. The mild cortical malformation is an important new finding, though it remains unknown whether these cortical abnormalities are due to the *KCNQ2* mutation or are secondary to the refractory seizures.

## Case Description

The patient was a pre-term born male [36 6/7 weeks, birth weight 3.4 kg (−1 SD)], head circumference 34 cm (−1.5 SD), and the second child of non-consanguineous parents. The pregnancy was uneventful, but the mother reported the occurrence of jittery fetal movements during the last trimester. At postnatal day 1, the child developed generalized and multifocal tonic seizures preceded by a high piercing cry and accompanied by desaturation and bradycardia. He subsequently developed left-sided limb and facial jerks accompanied again by desaturations. He showed mild dysmorphic features, retrognathia, and small hollow eyes. Neurological examination showed hypotonia, jitteriness, and absence of blinking and sucking reflex. At day 24, seizure frequency increased and non-invasive breathing support became necessary because of decreased spontaneous breathing. Physical examination showed persistent hypotonia and jitteriness, intermittent biking movements, absent sucking and search reflexes, and adducted thumbs.

Biochemical screening (serum electrolytes, glucose level, ammonia, and routine urine examination) was unremarkable. Metabolic investigation showed no abnormalities (including analysis for tyrosinemia, urea cycle defect and creatine synthesis defect, disturbances in purine, pyrimidine, and carbohydrate metabolism, and CSF lactate). Subsequent treatment with midazolam, folic acid, pyridoxal phosphate, levetiracetam, and phenobarbital was not successful.

**Figure 1:**
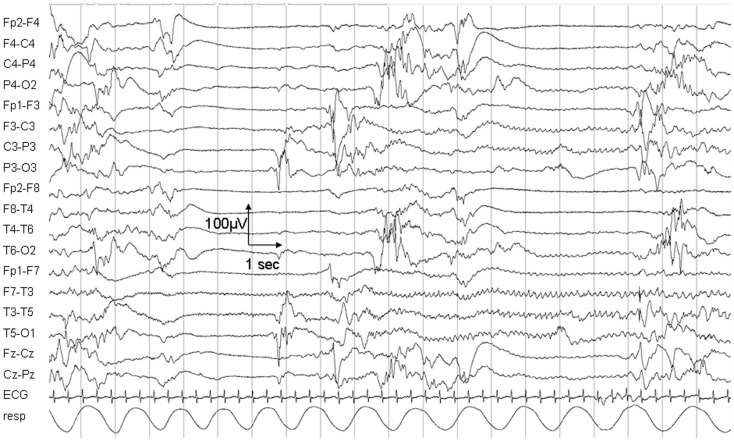
**Electroencephalogram recording on day 25**. The EEG shows a non-synchronized pattern with suppressions, multifocal sharp waves, and a focal epileptic seizure in the left hemisphere.

The child died on day 25 due to respiratory insufficiency during a status epilepticus. Electroencephalogram (EEG) at day 5 showed a non-synchronized immature background pattern with multiple focal sharp waves predominant over the right hemisphere and subclinical seizures lasting up to 40 s. At day 25, the EEG pattern was characterized by sharp waves and focal seizures isolated on the left hemisphere (Figure 1).

**Figure 2:**
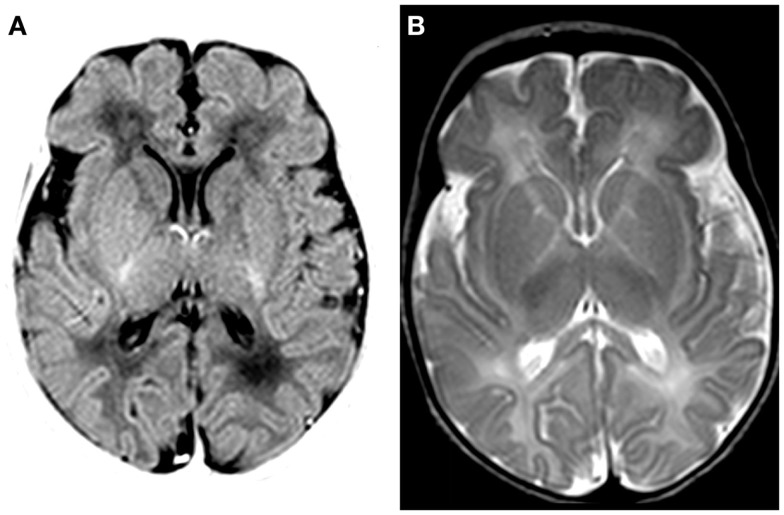
**MRI findings on day 7**. **(A)** T1-weighted MRI (inversion recovery) shows discrete swelling of the basal nuclei and thalamus. **(B)** T2-weighted MRI shows a decreased signal in basal nuclei and thalamus.

Magnetic resonance imaging (MRI; Siemens Sonata 1.5 T, Erlangen, Germany) at day 7 showed discrete swelling and hyperintensity of the basal nuclei (T1-weighted imaging; Figure 2). Diffusion-weighted images were normal (data not shown). MR proton spectroscopy showed normal concentrations of creatine, choline, and *N*-acetylaspartate and low lactate (data not shown). Post-mortem MRI examination showed no abnormalities (data not shown).

### Neuropathological methods

Autopsy was performed at the VU University medical center 12 h post-mortem. The results were compared with an age-matched control subject without significant confounding neuropathological findings. With the parents’ informed consent, the tissue was obtained and used in a manner in accordance with the Declaration of Helsinki.

The brain was fixed in buffered 4% formalin. After fixation, the cerebral hemispheres were cut in 10 coronal slices and the brainstem and cerebellum in 7 axial slices. Tissue samples were taken bilaterally from all cerebral lobes, the nucleus accumbens, lentiform nucleus, thalamus and subthalamus, hippocampus and cerebellar white matter with the nucleus dentatus, and cortex of the inferior semilunar lobe. The brainstem was sampled at the level of the midbrain, mid-pons and medulla oblongata. After embedding in paraffin, the tissue was sectioned at 6 μm and stained for Hematoxylin and Eosin (H&E), Nissl and Luxol fast blue-periodic acid Schiff according to standard methods. Additionally, tissue sections were incubated with antibodies against the following epitopes: glial fibrillary acidic protein (GFAP; Millipore, 1:1000), vimentin (DAKO, 1:1000); proteolipid protein (PLP; AbDSerotec, 1:3000), neuronal nuclear antigen (NeuN; Sigma, 1:500), microtubule-associated protein 2 (MAP2; Chemicon, 1:500), phosphorylated and non-phosphorylated neurofilament (SMI31 and SMI311, respectively; Covance, 1:1000), and human leukocyte antigen (HLA)-DP, -DQ, -DR (CR3/43; DAKO; 1:400). Briefly, sections were deparaffinized and rehydrated. Endogenous peroxidase activity was quenched by incubating the slides in 0.3% hydrogen peroxide in methanol. Heat-induced antigen retrieval was performed in citric acid (0.01 M, pH6) using microwave irradiation for 15 min on low setting. Tissue sections were incubated overnight with primary antibodies, and the staining was developed with diaminobenzidine tetrachloride. Sections were counterstained with hematoxylin, dehydrated, and mounted with polyvinyl alcohol medium with DABCO (Sigma). Negative controls by omitting the primary antibody were included in each experiment and were essentially blank. Tissue sections including the cortex and underlying white matter extending to the ventricles were obtained from both frontal lobes just ventral to the nucleus accumbens and stained with the neuronal-specific markers MAP2 and NeuN. Individual MAP2- and NeuN-immunoreactive neurons were counted manually on 10 images taken at 100× magnification of the deep white matter (>500 μm below the cortical boundary) and the density of ectopic neurons was expressed as number of immunoreactive cells per square millimeter. MAP2 and NeuN stained sections gave similar results.

**Figure 3:**
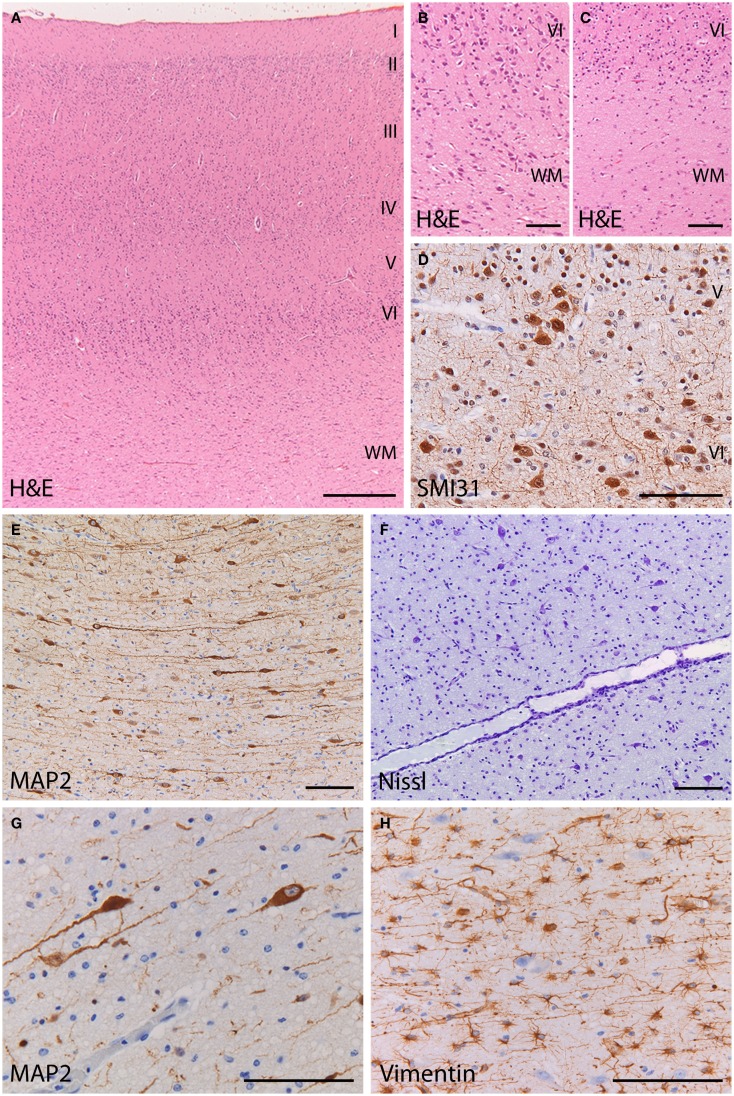
**Histopathology of the frontal cortex shows mild malformation of cortical development**. **(A)** H&E staining at low magnification shows a normal six-layered architecture of the cortex. **(B)** At high magnification of the cortex and cortical boundary, heterotopic neurons displaced to the white matter make it difficult to distinguish between the gray and white matter border (H&E). **(C)** In the frontal cortex of a control subject, the gray–white matter junction appears sharply demarcated (H&E). **(D)** Abnormal clusters of SMI31-immunoreactive neurons (arrow) are visible in the deep cortical layers. **(E,F)** The deep white matter (>500 mm from the cortical border) shows increased cellularity by heterotopic neurons [**(E)**, stain against the microtubule associated protein 2 (MAP2); **(F)** stain against extranuclear RNA identifying neurons]. **(G)** The heterotopic neurons in the deep white matter, identified by their MAP2-immunoreactivity, have normal morphology and immunophenotype. **(H)** The deep white matter harboring the heterotopic neurons is gliotic with numerous vimentin-positive reactive astrocytes. **(A)** 25×; **(B,C,E,F)** 100×; **(D,G,H)** 200×.

At gross examination, the brain had a normal structure with preservation of the neocortical gyral pattern. Microscopic examination of the cerebral cortex showed a six-layered architecture without obvious dyslamination or columnar disorganization (Figure 3A) (see additional materials for details). Occasional neuronal clustering in cortical layers V and VI was observed in the frontal lobes (Figure 3D). The interface between cortex and underlying white matter was diffusely blurred by the presence of heterotopic neurons displaced to the white matter, as compared to normal (Figures 3B,C). Numerous individual neurons were also found in deep white matter locations (mean 47 MAP2^+^ neurons/mm^2^; mean 6 MAP + neurons/mm^2^ in the age-matched control) (Figures 3E,F). These heterotopic neurons had normal morphology and immunophenotypic profile (Figure 3G). No dysmorphic neurons or balloon cells were seen. The cortex and underlying white matter displayed intense reactive gliosis (Figure 3H). The white matter contained virtually no mature myelin, as expected for the age. No hippocampal sclerosis or other lesions were found. No abnormalities were found in the cerebellum, including cortex and dentate nucleus. These features are consistent with malformation of cortical development (mMCD) (1–3).

Genetic analysis disclosed a *de novo* heterozygous missense mutation (c.643G < A; p.Gly215Arg) in the *KCNQ2* gene (reference sequence NM_172107.2).

## Background

Neonatal convulsions are clinical manifestations in a heterogeneous group of disorders with different etiology and outcome.

Benign familial neonatal convulsion (BFNC) is characterized by a benign course with spontaneous remission and a moderate risk for development of epilepsy later in life (4).

Benign familial neonatal convulsion is defined by the following criteria: occurrence of frequent brief seizures on or after the second day of life, which disappear spontaneously within a few weeks; positive family history with an autosomal dominant inheritance pattern; exclusion of other causes of neonatal seizures; and normal physical examination and subsequent neurodevelopment (4, 5). BFNC is caused by mutations in two genes, *KCNQ2* and *KCNQ3* (6, 7). In addition, *KCNQ2* mutations have been found in a few patients with refractory neonatal seizures similar to the Ohtahara syndrome (4). In our patient, genetic analysis revealed a *de novo* heterozygous missense mutation in the *KCNQ2* gene, which encodes a subunit of a voltage-gated potassium channel.

In affected families with BFNC, the individuals who later presented with delayed psychomotor development or mental retardation showed mutations located on the *KCNQ2* gene (1) suggesting an association between *KCNQ2* mutations and encephalopathy. EEG, MRI data, as well as mutation analysis have been described in other *KCNQ2* cases (8). This case report provides EEG, MRI, genetic analysis, and neuropathological data. Neuropathological investigation revealed a mild mMCD (1–3). The new finding of this study is the combination of a *KCNQ2* mutation and mMCD.

## Discussion

There is significant literature on potassium-channel gene mutations in the brain and various types of epilepsy (9–11). There are many animal models to study potassium channels and mechanism for hyperexcitability and correlates of genetic mutations in the KCNQ2 gene (4, 12, 13). Literature shows increased seizure susceptibility in rats with induced nodular heterotopia. These heterotopic neurons showed inactivated expression of Kv4.2 subunits in cultured cells (13). Potassium channels play critical roles in modulating neuronal excitability and mutations in these channels can result in severe epileptic phenotypes, as we describe in our study.

*KCNQ2* encodes a subunit of a voltage-gated potassium channel. It is expressed in the central nervous system from 22 weeks gestational age onward, where it inhibits neuronal excitability (14). *KCNQ2* mutations are associated with different clinical phenotypes, including typical BFNC (5, 9) and neonatal or early infantile epilepsy (BFNIS) (7). Changes in the *KCNQ2* gene have been identified in 60–70% of families with BFNC (7).

Benign familial neonatal convulsion seizures are characterized by a tonic component followed by a range of motor phenomena associated with ocular symptoms, dyspnea, and autonomic signs. Seizures are usually brief but can evolve into status epilepticus. Typically, BFNC has a good outcome with most patients being seizure-free after 6 months. However, several patients with a severe encephalopathy phenotype of *KCNQ2*-related neonatal seizures have also been reported (14, 15).

Most of the reported *KCNQ2* encephalopathies were diagnosed as Ohtahara syndrome showed a burst suppression EEG pattern and infrequently involuted to West syndrome with poor developmental outcome (8).

*KCNQ2* encephalopathy is characterized by onset of intractable seizures in the first week of life with a prominent tonic component in the first week of life. In line with a previous study (14), the mother of our patient reported jittery fetal movements in the last trimester of pregnancy, suggesting that seizures onset may be prenatal. MRI findings include discrete signal hyperintensity of the basal nuclei and thalamus, which are different from those typically observed in patients with hypoxic–ischemic encephalopathy (7). Although seizures resolved before 3 years of age, surviving patients were left with intellectual disability and motor impairment (14).

Our patient carried a *de novo* mutation in the *KCNQ2* gene. The resulting p.Gly215Arg amino-acid change is located in the S4 transmembrane domain, which acts as the voltage-sensor of the potassium-channel subunit Kv7.2. Mutation in this domain was also found in two patients in the series of Weckhysen et al. (14). It is hypothesized that mutations in this area are associated with the more severe phenotype of encephalopathy (14). Whether KCNQ2 encephalopathy patients may benefit from potassium-channel openers remains to be established (4). They could benefit from carbamazepine, stabilizing the inactive state of voltage-gated sodium channels, followed by retigabine, which increases the potassium current through KCNQ2 channels (16). The electroclinical and early radiological features of our patient were consistent with a *KCNQ2* encephalopathy. However, the seizure frequency progressively increased despite a broad anti-epileptic regimen and the child died during a status epilepticus. As already reported, the EEG showed a non-synchronized pattern with multifocal sharp waves and multifocal neonatal seizures. The electroclinical features closely resembled the Ohtahara syndrome, in line with the severity of the epileptic encephalopathy. These burst suppression patterns with associated interictal multifocal spikes and polyspikes in association with the specific neonatal clinical epileptiform activity must be taken into account in the diagnostic work-up of neonatal encephalopathies, which also includes *KCNQ2* testing (16, 17).

The association of *KCNQ2* mutations and refractory epilepsia with neuropathological abnormalities has not been described. Studies confirm an association between *KCNQ2* mutation and neonatal onset epileptic encephalopathy (18). The new finding of this study is the combination of a KCNQ2 mutation and mMCD.

The neuropathological findings were consistent with mMCD. mMCD is characterized by neuronal clusters or an excess of single neurons with normal morphology in the deep white matter in the absence of other lesions (1, 2, 19). White matter neurons are a normal finding in the mature brain (2). A proportion of these cells are likely to be remnants of subplate cells, important for the establishment of thalamo-cortical pathways during development. In the mature brain, white matter neurons include excitatory as well as inhibitory interneurons possibly functionally integrated into cortical circuits (20). However, numerous studies confirm excessive numbers of white matter neurons in epilepsy patients (21). The finding of heterotopic neurons in our patient might explain the persistence of intractable seizures with the fatal course. Possible explanations for a microscopic neuronal heterotopia are either developmental abnormality (i.e., heterotopic neurons following failure of normal migration), a maturational abnormality (abnormal persistence of subplate neurons following corticogenesis) or neurogenesis, possibly influenced by seizures.

In conclusion, *KCNQ2* mutations should be considered in neonates with refractory epilepsy of unknown cause. The mild cortical malformation is an important new finding, though it remains unknown whether the observed cortical abnormalities are causally related to the *KCNQ2* mutation or are secondary to the refractory seizures.

## Author Contributions

Charlotte Dalen Meurs-van der Schoor and R. Jeroen Vermeulen were responsible for conducting the study, drafted, and revised the manuscript critically for intellectual content and performed the literature search. R. Jeroen Vermeulen and Mirjam van Weissenbruch were responsible for collection of samples and clinical data. Marjan van Kempen was responsible for the genetic analysis/sequencing and data collection. Eleonora Aronica and Marianna Bugiani performed the neuropathological analysis, designed the neuropathological methods, and revised the manuscript. Hanneke Ronner contributed to the neurophysiological data analysis (EEG). All authors participated in the revision of the draft and gave their final approval of the version to be published. All authors agree to be accountable for all aspects of the work in ensuring that questions related to the accuracy or integrity of any part of the work are appropriately investigated and resolved.

## Conflict of Interest Statement

The authors have no conflicts of interest to disclose. All co-authors have been substantially involved in the study and preparation of the manuscript; all authors have read and approved the submitted manuscript. Information and images herein are presented with appropriate consent obtained and with details removed that might potentially reveal the identity.
